# Phloretin ameliorates hepatic steatosis through regulation of lipogenesis and Sirt1/AMPK signaling in obese mice

**DOI:** 10.1186/s13578-020-00477-1

**Published:** 2020-09-29

**Authors:** Chian-Jiun Liou, Shu-Ju Wu, Szu-Chuan Shen, Li-Chen Chen, Ya-Ling Chen, Wen-Chung Huang

**Affiliations:** 1 Department of Nursing, Division of Basic Medical Sciences, Research Center for Chinese Herbal Medicine, Chang Gung University of Science and Technology, No.261, Wenhua 1st Rd., Guishan Dist., Taoyuan City, 33303 Taiwan; 2 Division of Allergy, Asthma, and Rheumatology, Department of Pediatrics, Chang Gung Memorial Hospital, Linkou, Guishan Dist., Taoyuan City, 33303 Taiwan; 3 Department of Nutrition and Health Sciences, Research Center for Chinese Herbal Medicine, Chang Gung University of Science and Technology, No.261, Wenhua 1st Rd., Guishan Dist., Taoyuan City, 33303 Taiwan; 4 Aesthetic Medical Center, Department of Dermatology, Chang Gung Memorial Hospital, Linkou, Guishan Dist., Taoyuan, 33303 Taiwan; 5 Graduate Program of Nutrition Science, National Taiwan Normal University, 88 Ting-Chow Rd, Sec 4, Taipei City, 11676 Taiwan; 6 School of Nutrition and Health Sciences, Taipei Medical University, 250 Wu-Hsing Street, Taipei City, 11031 Taiwan; 7 Graduate Institute of Health Industry Technology, Research Center for Food and Cosmetic Safety, Research Center for Chinese Herbal Medicine, College of Human Ecology, Chang Gung University of Science and Technology, No.261, Wenhua 1st Rd., Guishan Dist., Taoyuan City, 33303 Taiwan

**Keywords:** HepG2, Lipogenesis, Lipolysis, Phloretin, non-alcoholic fatty liver disease

## Abstract

**Background:**

Phloretin is isolated from apple trees and could increase lipolysis in 3T3-L1 adipocytes. Previous studies have found that phloretin could prevent obesity in mice. In this study, we investigated whether phloretin ameliorates non-alcoholic fatty liver disease (NAFLD) in high-fat diet (HFD)-induced obese mice, and evaluated the regulation of lipid metabolism in hepatocytes.

**Methods:**

HepG2 cells were treated with 0.5 mM oleic acid to induce lipid accumulation, and then treated with phloretin to evaluate the molecular mechanism of lipogenesis. In another experiment, male C57BL/6 mice were fed normal diet or HFD (60% fat, w/w) for 16 weeks. After the fourth week, mice were treated with or without phloretin by intraperitoneal injection for 12 weeks.

**Results:**

Phloretin significantly reduced excessive lipid accumulation and decreased sterol regulatory element-binding protein 1c, blocking the expression of fatty acid synthase in oleic acid-induced HepG2 cells. Phloretin increased Sirt1, and phosphorylation of AMP activated protein kinase to suppress acetyl-CoA carboxylase expression, reducing fatty acid synthesis in hepatocytes. Phloretin also reduced body weight and fat weight compared to untreated HFD-fed mice. Phloretin also reduced liver weight and liver lipid accumulation and improved hepatocyte steatosis in obese mice. In liver tissue from obese mice, phloretin suppressed transcription factors of lipogenesis and fatty acid synthase, and increased lipolysis and fatty acid β-oxidation. Furthermore, phloretin regulated serum leptin, adiponectin, triglyceride, low-density lipoprotein, and free fatty acid levels in obese mice.

**Conclusions:**

These findings suggest that phloretin improves hepatic steatosis by regulating lipogenesis and the Sirt-1/AMPK pathway in the liver.

## Background

Refined foods and junk food increase the prevalence of obesity, which is a risk factor for chronic diseases, including cardiovascular disease, atherosclerosis, type 2 diabetes, and cancer [1]. Obesity not only increases the lipid accumulation of visceral fat tissue, but also induces excessive lipid accumulation in the liver and causes fatty liver disease [2]. Non-alcoholic fatty liver disease (NAFLD) is a common hepatic disease, and the development of NAFLD is considered to be a complex metabolic syndrome of abnormal liver metabolism. Patients with obesity or type 2 diabetes also often suffer from NAFLD. Epidemiological studies indicate that approximately 75% of overweight and obese patients in developed and developing countries have NAFLD [3]. The initial development of NAFLD is excessive lipid accumulation in the liver, which is known as liver steatosis. A total of 5–20% of patients with steatosis will develop a more severe non-alcoholic steatohepatitis (NASH), which is characterized by liver inflammation, fibrosis, and tissue damage [4]. Patients with NASH who do not receive medical treatment and regulate their unhealthy lifestyle and eating habits will develop irreversible liver fibrosis, cirrhosis, liver failure, and liver cancer [5]. In addition, NAFLD is thought to increase the risk of cardiovascular disease (coronary atherosclerosis) [6]. Therefore, treatment of NAFLD could attenuate the incidence of many chronic diseases.

The pathological mechanism of NAFLD is not fully understood. Hepatic steatosis is caused by increased lipid accumulation in the liver and reduced lipid breakdown [6]. However, excessive fat accumulation will also induce liver lipid toxicity, oxidative stress, and inflammation, leading to hepatocyte damage and death [7]. Therefore, excessive accumulation of oil droplets will interfere with the physiological function of the liver. With excessive intake of a high-calorie diet, via digestion and metabolism, high-energy foods will be converted into triglycerides (TGs) in the liver and adipocytes [3]. In the lipid synthesis pathway, activation of transcription factors CCAAT/enhancer-binding protein (C/EBP) and sterol regulatory element binding protein 1c (SREBP-1c) regulates the expression of fatty acid synthase (FAS) to increase the lipid synthesis reaction [8]. Therefore, the expression of SREBP-1c and FAS can be higher in the liver or visceral adipose tissue of obese individuals than normal-weight persons.

Many studies have pointed out that accelerating lipid breakdown in the liver of an obese individual would decrease lipid accumulation in the liver and improve NAFLD [9]. In the liver, TG decomposition requires lipases, including adipose triglyceride lipase (ATGL) and hormone-sensitive lipase (HSL), which can break down TGs to produce free fatty acids and glycerol [10]. ATGL-knockout mice cannot lose significant weight under the conditions of proper exercise and calorie restriction [11]. Obviously, the activation of lipase could assist and improve NAFLD. However, excessive free fatty acids from the breakdown of TGs need to be converted to produce energy by β-oxidation [9]. Therefore, promoting lipolysis can regulate lipid metabolism and improve NAFLD towards normal liver function.

In liver, muscle, and adipose tissue, the AMPK signaling pathway can contribute to maintaining an energy balance and regulate lipid metabolism [12]. Activated AMPK can inhibit the expression of lipid synthesis-related proteins, such as SREBP1, FAS, and CD36, and can also increase expression in the fatty acid β-oxidation pathway and related enzymes (e.g., carnitine palmitoyltransferase 1 [CPT-1] and peroxisome proliferator-activated receptor alpha [PPARα]) to decrease lipid accumulation [13]. Previous studies have shown that AMPK activation can protect liver cells from oxidative damage and inflammation, and can inhibit apoptosis and improve the development of NAFLD [14]. AMPK phosphorylation can also stimulate acetyl-CoA carboxylase (ACC) phosphorylation, reducing lipid biosynthesis [13]. Sirtuin 1 (Sirt1) is an NAD^+^-dependent deacetylase that can contribute to regulating intracellular NAD^+^ levels for maintaining cellular energy balance [15]. In obese mice, adipocytes and liver cells accumulate excess lipid, which inhibits the activity of Sirt1 and AMPK, inactivating the Sirt1/AMPK pathway and aggravating the development of NAFLD [16]. Resveratrol is a Sirt1 enhancer. The treatment of obese mice with resveratrol could induce the expression of Sirt1 and phosphorylation of AMPK, regulating the molecular pathways of lipid and glucose metabolism in the liver [16, 17]. Therefore, stimulating the activation of the Sirt1/AMPK pathway will reduce lipid accumulation in the liver and improve hepatic steatosis in obese and overweight individuals.

Clinically effective drugs for preventing or treating NAFLD are still lacking [18]. Plant extracts or natural products have been investigated extensively for preventing or improving obesity and NAFLD [19, 20]. Phloretin is isolated from apple trees [21]. Our previous studies found that phloretin can inhibit lipid accumulation in 3T3-L1 adipocytes via inhibition of adipogenesis-related transcription factors and promotion of AMPK phosphorylation [22]. Phloretin can also reduce inflammatory adipokines and increase adiponectin expression in LPS or LPS/CoCl_2_-stimulated 3T3-L1 adipocytes [23]. Other studies have found that phloretin can prevent weight gain and regulate blood glucose in high-fat diet (HFD)-induced obese mice [24]. However, the beneficial effects of phloretin on NAFLD are unclear. In the present study, we investigated whether phloretin regulates lipogenesis in HepG2 hepatocytes in vitro, ameliorates NAFLD, and modulates the molecular mechanism underlying lipid metabolism in HFD-induced obese mice.

## Materials and methods

### Cell culture and induced fatty liver cells

The HepG2 hepatocyte cells were cultured in DMEM medium supplemented with 1% penicillin–streptomycin solution and 10% fetal bovine serum. Phloretin (isolated from apple wood, purity ≥ 99% by HPLC) was purchased from Sigma–Aldrich (St. Louis, MO, USA). Phloretin were dissolved in DMSO, and for all cell experiments the final culture concentration of DMSO was < 1%. For cell viability assays, HepG2 cells were treated with various concentrations of phloretin or oleic acid for 48 h to measure cell viability using MTT solution (Sigma) as described previously [25]. The culture plate was treated with isopropanol to evaluate cell viability using a microtiter plate reader (Multiskan FC, Thermo Fisher Scientific) at an absorbance of 550 nm. To detect lipid accumulation in hepatocytes, HepG2 cells were incubated with 0.5 mM oleic acid to stimulate lipid accumulation for 48 h. Cells were treated with vehicle (0.1% DMSO) or phloretin (3–30 μM) for 24 h to detect the molecular mechanism of lipid metabolism. In other cellular experiments, phloretin-treated HepG2 cells were treated with the AMPK inhibitor compound C (Sigma) to evaluate the molecular expression of lipid metabolism.

### Oil Red O staining

HepG2 cells were incubated with 0.5 mM oleic acid for 48 h and then treated with or without phloretin for 24 h. The culture plate was washed and cells fixed with formalin. Hepatocytes were stained with Oil Red O solution to observe the distribution of oil droplets in liver cells as described previously [26]. Finally, hepatocytes were treated with isopropanol to assay lipid accumulation using a microplate reader (Multiskan FC) at an absorbance of 490 nm.

### Western blot analysis

Proteins were separated by 8–10% SDS–PAGE and transferred to polyvinylidene difluoride membrane. The membrane was incubated with specific antibodies overnight at 4 °C, followed by secondary antibodies at room temperature for 1 h. Protein signals were visualized using luminol/enhancer solution and the BioSpectrum 600 system (UVP, Upland, CA, USA). The specific primary antibodies were AMPKα, phosphorylated AMPKα (pAMPKα), CPT-1, CPT2, FAS, Serbp-1c, Sirt1 (Cell Signaling Technology, MA, USA), β-actin (Sigma), ATGL, acetyl CoA carboxylase-1 (ACC-1), phosphorylated-ACC-1 (pACC-1), C/EBPβ, HSL, phosphorylated HSL (pHSL), and PPAR-α (Abcam, Cambridge, MA, USA).

### Animals and treatments

Four-week-old male C57BL/6 mice (National Laboratory Animal Center, Taiwan) were randomly divided into four groups of 10. HFD based on research diet TestDiet 58Y1 (Purina TestDiet, St. Louis, MO, USA) containing 23.1% protein, 34.9% fat, and 25.8% carbohydrates, and fat supplied 60% of energy. The normal control group (N) and HFD control group (HFD) were fed a standard chow diet or HFD, respectively, and administered DMSO solution by intraperitoneal injection. The PT10 and PT20 groups were fed a HFD and administered 10 mg/kg and 20 mg/kg phloretin by intraperitoneal injection, respectively. The HFD, PT10, and PT20 groups were fed a HFD for 4 weeks. All mice were treated with 50 μl DMSO or phloretin (dissolved in DMSO) twice a week for 12 weeks (Fig. 2a). Dietary intake was recorded per day and body weight measured weekly. Food intake (the weight of consumed food (g) × calories in the diet) was recorded each day. Animal experiments were approved by the Laboratory Animal Care Committee of Chang Gung University of Science and Technology (IACUC approval number: 2015–019).

### Biochemical analysis

Mice were anesthetized with isoflurane and blood collected via the orbital vascular plexus. The blood was centrifuged and the serum collected to detect the free fatty acids and low-density lipoprotein (LDL) using a fatty acid quantitation kit and LDL quantitation kit (Sigma), respectively, according to the manufacturer’s protocol as described previously [26]. The serum levels of glutamate oxaloacetate transaminase (GOT), glutamate pyruvate transaminase (GPT), total cholesterol (TC), high-density lipoprotein (HDL), and total TGs were measured using the biochemical analyzer (DRI-CHEM NX500, Fuji, Tokyo, Japan). The day before the end of the experiment, mice fasted for 16 h and were administered glucose by intraperitoneal injection to assay blood glucose levels using the biochemical analyzer (Fuji). Blood insulin was detected using the Insulin EIA Kit (Cayman, Ann Arbor, Michigan, USA) according to the manufacturer’s protocol. Liver glycogen was detected using the Glycogen Assay Kit (Cayman) according to the manufacturer’s protocol, and the glycogen levels were measured using a microplate reader (Multiskan FC) at an absorbance of 570 nm.

### Histological analysis

Liver tissues were removed and embedded in paraffin. All tissues were cut into 6-μm sections and stained using hematoxylin and eosin (HE) solution as described previously [27]. Glycogen expression in the liver tissue was detected by periodic acid-Schiff (PAS) solution. All biopsy specimens were observed under a light microscope (Olympus, Tokyo, Japan) and an NAFLD score evaluated as described previously [28]. Furthermore, epididymal and inguinal adipose tissue were removed, weighed, and fixed in formalin solution. Next, adipose tissues were embedded in paraffin as described previously [26]. In briefly, all adipose tissues were cut into 6-μm sections and stained with HE solution to observe and take a photograph with a light microscope (Olympus). Furthermore, the images of adipose tissue choose five fat cells to calculate the cell area using cellSens Standard software (Olympus).

### Real-time PCR

Liver tissues in TRI reagent (Sigma) were homogenized using a homogenizer (FastPrep-24, MP Biomedicals, Santa Ana, CA, USA). Samples were centrifuged and the supernatant collected to extract total RNA. Next, cDNA was synthesized using the cDNA Synthesis Kit (Life Technologies, Carlsbad, CA) as described previously [29]. Using fluorescently labeled SYBR Green treated with DNA sample solutions, we amplified specific gene expression using a spectrofluorometric thermal cycler (iCycler; Bio-Rad Laboratories, Hercules, CA, USA).

### Statistical analysis

Statistical analyses were performed by one-way analysis of variance (ANOVA) and a Dunnett post hoc test. All data were investigated in at least three independent experiments, and data are presented as the mean ± SEM. *P* < 0.05 was considered significant.

## Results

### Cell viability of phloretin in HepG2 cells

The cytotoxicity of phloretin in HepG2 cells was determined using the MTT assay. Phloretin did not demonstrate significant cytotoxic effects at a concentration ≤ 50 μM, and subsequent experiments used phloretin at 3–30 μM concentrations for all cell experiments (data not shown). We also determined that oleic acid concentrations ≤ 0.5 mM did not significantly affect cell viability in HepG2 cells (data not shown).

### Phloretin attenuated lipid accumulation in HepG2 cells

**Fig. 1:**
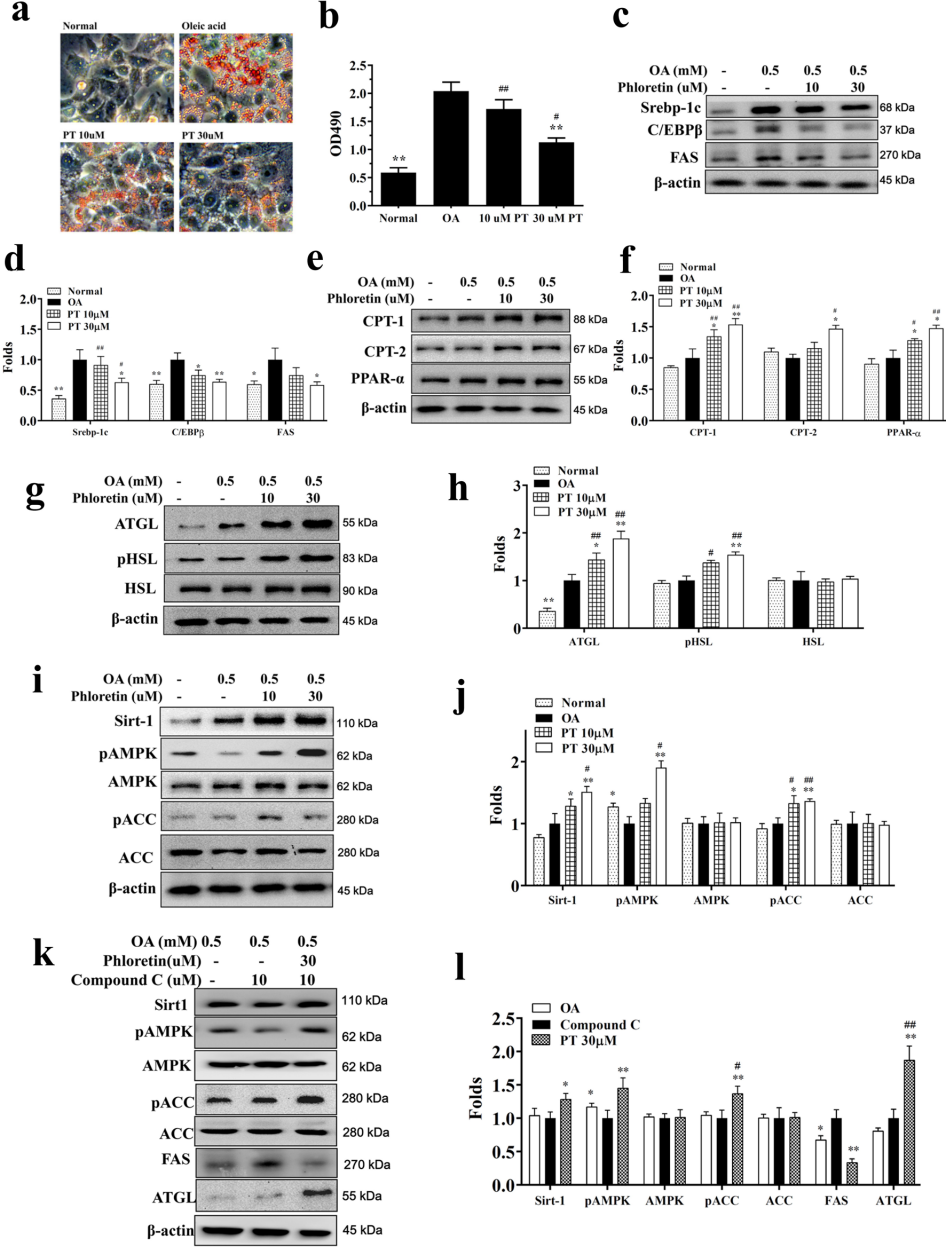
Phloretin (PT) reduced lipid accumulation in HepG2 cells. **a** Oil Red O staining revealed lipid accumulation. **b** HepG2 cells were treated with isopropanol and lipid accumulation measured using the absorbance at OD 490 nm. Effects of phloretin (PT) on lipid metabolism in oleic acid (OA)-induced HepG2 cells. **c** The expression of transcription factors and FAS and **d** the fold expression were measured relative to β-actin. **e** β-oxidation and **f** the fold expression were measured relative to β-actin. **g** Lipolysis was detected by Western blot and **h** the fold expression measured relative to the expression of β-actin. **i** The Sirt1/AMPK pathway and **j** fold expression measured relative to β-actin. Data are presented as mean ± SEM; *P < 0.05, **P < 0.01 compared to the OA group. ^#^*P* < 0.05, ^##^*P* < 0.01 compared to the without treated-OA group. Furthermore, **k** HepG2 cells were treated with 0.5 mM OA for 48 h, followed by 30 μM phloretin with or without an AMPK inhibitor (compound c) for 24 h. **l** The fold expression was measured relative to β-actin. Three independent experiments were analyzed and the data presented as the mean ± SEM. *P < 0.05, **P < 0.01 compared to the compound c group. ^#^*P* < 0.05, ^##^*P* < 0.01 compared to the OA group

Based on Oil Red O staining, phloretin decreased lipid droplets compared to oleic acid–induced HepG2 cells (Fig. 1a). Using isopropanol to treat hepatocytes confirmed that phloretin significantly attenuated lipid accumulation in oleic acid–treated hepatocytes (Fig. 1b).

### Effect of phloretin on lipid metabolism in hepatocytes

Protein assay demonstrated that phloretin significantly decreased Srebp-1c, C/EBPβ, and FAS expression compared to oleic acid-induced HepG2 cells (Fig. 1c, d). Phloretin could increase ATGL, pHSL, CPT-1, CPT-2, and PPARα production in oleic acid-induced HepG2 cells (Fig. 1e–h). Phloretin also significantly increased Sirt-1, phosphorylation of ACC, and phosphorylation of AMPK in a concentration-dependent manner compared to oleic acid-treated HepG2 cells (Fig. 1i, j). In oleic acid-induced HepG2 cells co-treated with 30 μM phloretin and compound C, phloretin restored phosphorylated AMPK, phosphorylated ACC, Sirt1, and ATGL, and inhibited FAS expression (Fig. 1k, l).

### Phloretin reduced HFD-induced obesity in mice

**Fig. 2:**
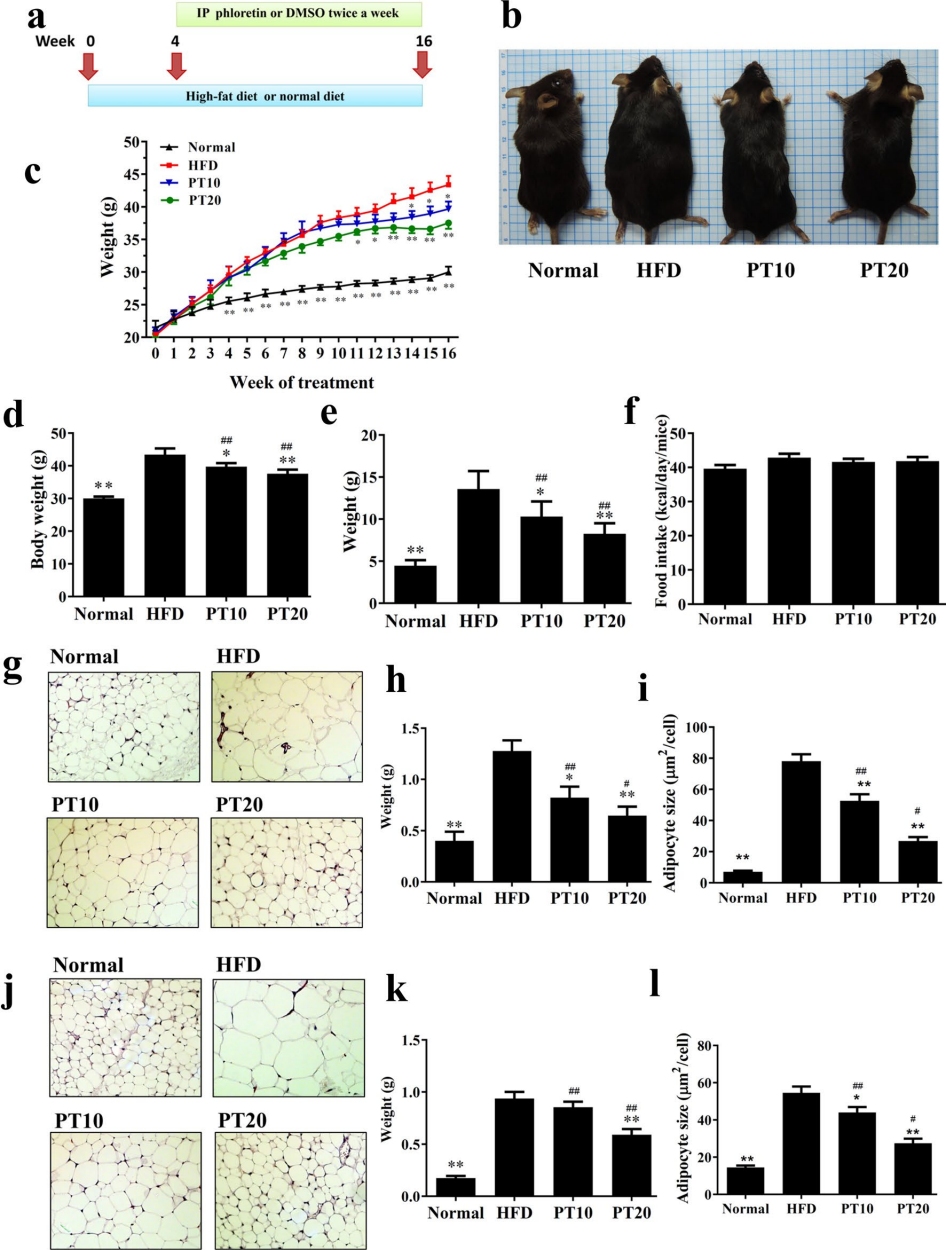
Phloretin (PT) reduced body weight in mice with HFD-induced obesity. **a** Male mice were fed a HFD (containing 60% fat) for 16 weeks and administered DMSO, 10 mg/kg phloretin (PT10), or 20 mg/kg phloretin (PT20) via intraperitoneal injection (I.P.) twice a week from week 4 to week 16. **b** Appearance of the mice. **c** Body weight was measured for 16 weeks. **d** Body weight was measured in the last week and **e** weight gain measured from the fourth week to the 16th week. **f** Food intake was monitored each day. Phloretin (PT) reduced the epididymal and inguinal adipose tissue weight in HFD-induced obese mice. **g** HE staining of epididymal adipose tissue (200 × magnification). **h** Weight of epididymal adipose tissue. **i** Size of adipocytes in epididymal adipose tissue. **j** HE staining of inguinal adipose tissue (200 × magnification). **k** Weight of inguinal adipose tissue. **l** Size of adipocytes in inguinal adipose tissue. Data are presented as mean ± SEM; n = 10. *P < 0.05, **P < 0.01 compared to HFD group. ^#^*P* < 0.05, ^##^*P* < 0.01 compared to the Normal group

We observed the appearance of the mice at the end of the experiments and found that HFD-induced obese mice were larger and fatter than normal mice (Fig. 2b). Interestingly, PT10 and PT20 mice had significant weight loss compared to HFD mice (39.69 ± 0.36 g, *P* < 0.05 and 37.55 ± 0.40 g, *P* < 0.01 vs. 43.40 ± 0.60 g, respectively; Fig. 2c, d). For obese mice treated with phloretin for 12 weeks, the weight gain in the PT10 and PT20 groups was significantly less than in the HFD group (PT10: 10.52 ± 0.73 g, *P* < 0.05; PT20: 8.39 ± 0.50 g, *P* < 0.01; HFD: 13.80 ± 0.78 g; Fig. 2e). The PT10 and PT20 groups did not have altered food intake compared to the HFD group (Fig. 2f).

### Phloretin reduced the weight of adipose tissue in obese mice

Using biopsy specimens, we found that phloretin significantly reduced the epididymal (Fig. 2g, h) and inguinal (Fig. 2j, k) adipose tissue weight compared to HFD mice. Phloretin also significantly decreased adipocyte size in the epididymal (Fig. 2i) and inguinal (Fig. 2l) adipose tissue compared to HFD mice.

### Phloretin attenuated liver steatosis in obese mice

**Fig. 3:**
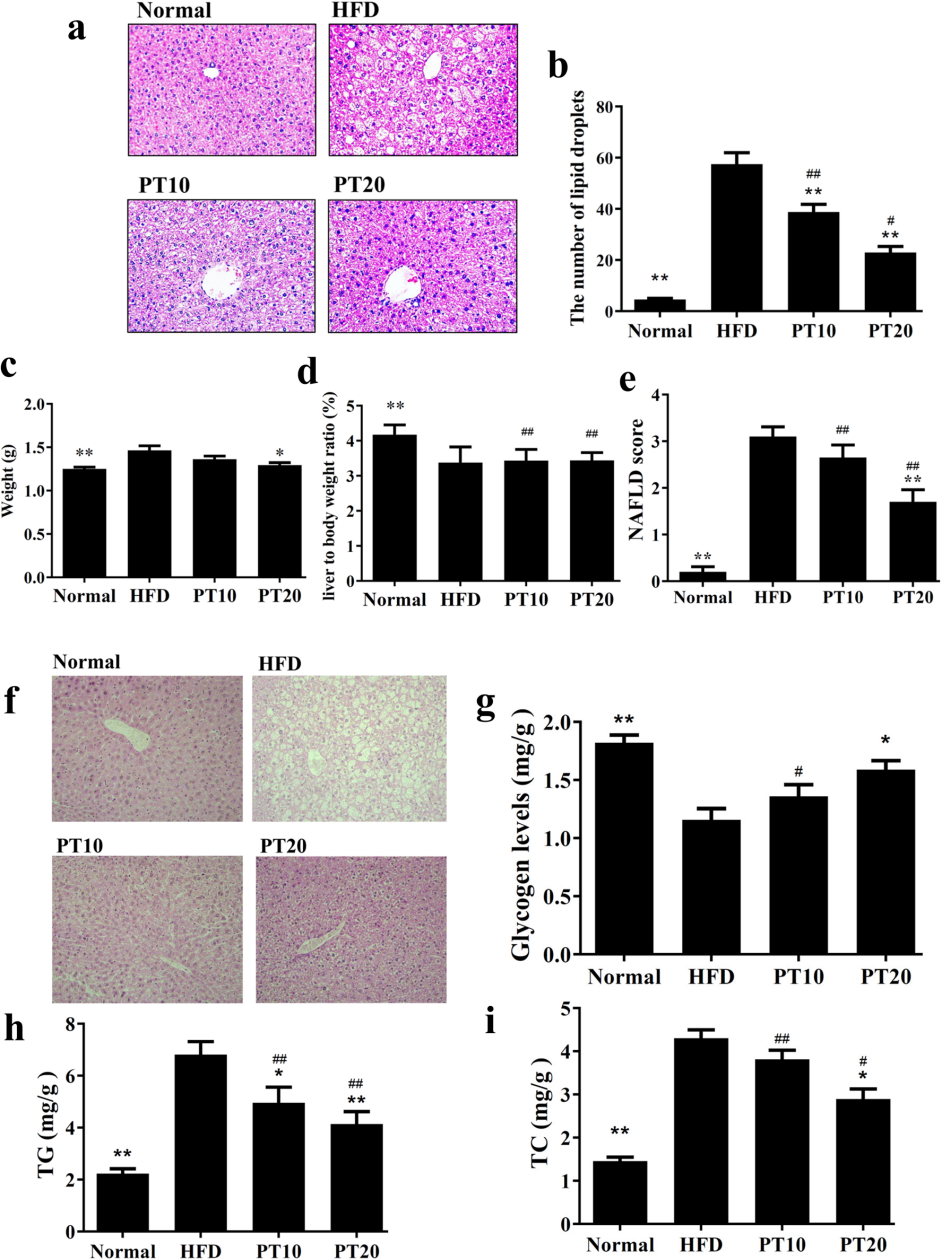
Phloretin (PT) ameliorated hepatic steatosis in HFD-induced obese mice. **a** HE staining of liver tissues (200 × magnification). **b** Calculated number of lipid droplets in liver tissue. **c** Liver weight, **d** the ratio of liver weight to body weight and **e** NAFLD scores based on HE staining. **f** PT modulated the glycogen distribution based on PAS staining in the liver (200 × magnification). **g** PT increased glycogen levels and **h** modulated triglyceride (TG) and **i** total cholesterol (TC) levels in the liver tissue. Three independent experiments were analyzed and the data presented as the mean ± SEM; n = 10. *P < 0.05, **P < 0.01 compared to HFD-induced obesity. ^#^*P* < 0.05, ^##^*P* < 0.01 compared to the Normal group

In HFD-induced obese mice, we found many fat vacuoles and lipid droplets distributed in the liver tissue. Our experiment found that obese mice treated with phloretin had significantly decreased fat vacuoles and fewer lipid droplets compared to HFD-induced obese mice (Fig. 3a, b). We also found that phloretin reduced the liver weight compared to obese mice (Fig. 3c). However, obese mice treated with phloretin did not decrease the liver to body weight ratio compared to HFD-induced obese mice (Fig. 3d). Furthermore, HFD mice treated with phloretin had significantly decreased NAFLD scores than the HFD group (Fig. 3e). PAS staining demonstrated that phloretin increased the glycogen distribution in liver tissue compared to HFD-induced obese mice (Fig. 3f). Thus, phloretin significantly recovered the glycogen levels (Fig. 3g) and reduced the levels of TC and TG (Fig. 3h-i) in the livers of mice with HFD-induced obesity.

### Phloretin regulated adipogenesis in liver tissue

**Fig. 4:**
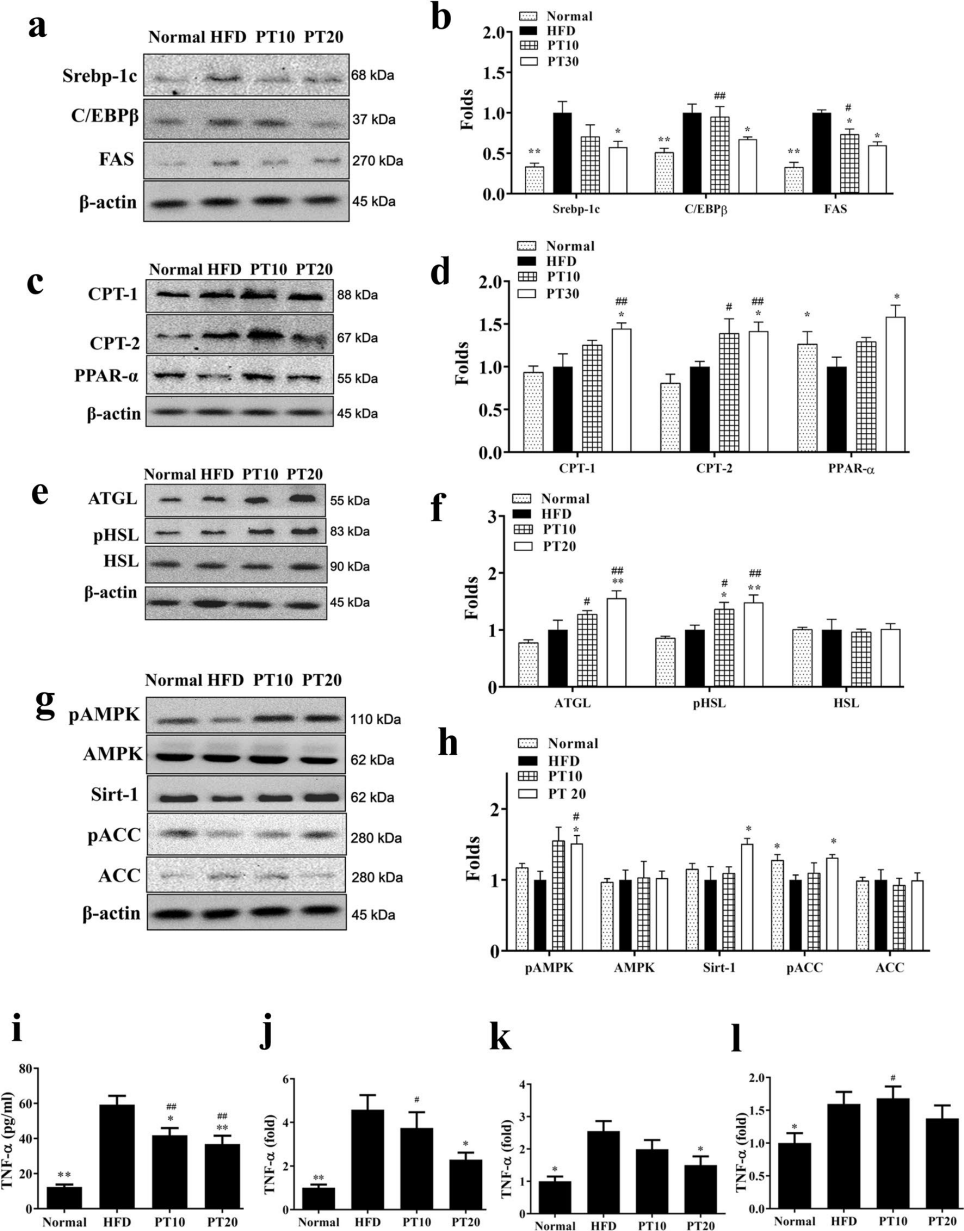
Effects of phloretin (PT) on lipid metabolism in mouse liver tissue. **a** The expression of transcription factors and FAS and **b** the fold expression were measured relative to β-actin. **c** β-oxidation and **d** the fold expression were measured relative to β-actin. **e** Lipolysis was detected by Western blot and **f** the fold expression measured relative to β-actin. **g** The Sirt1/AMPK pathway and **h** the fold expression measured relative to β-actin. Effects of phloretin (PT) on TNF-α -expression. **i** TNF-α levels in serum from mice and **j** gene expression in the liver, **k** epididymal adipose tissue, and **l** inguinal adipose tissue. Fold-changes in expression were measured relative to β-actin expression levels (internal control). Three independent experiments were analyzed and the data presented as the mean ± SEM; n = 10. *P < 0.05, **P < 0.01 compared to HFD-induced obesity. ^#^*P* < 0.05, ^##^*P* < 0.01 compared to the Normal group

**Fig. 5:**
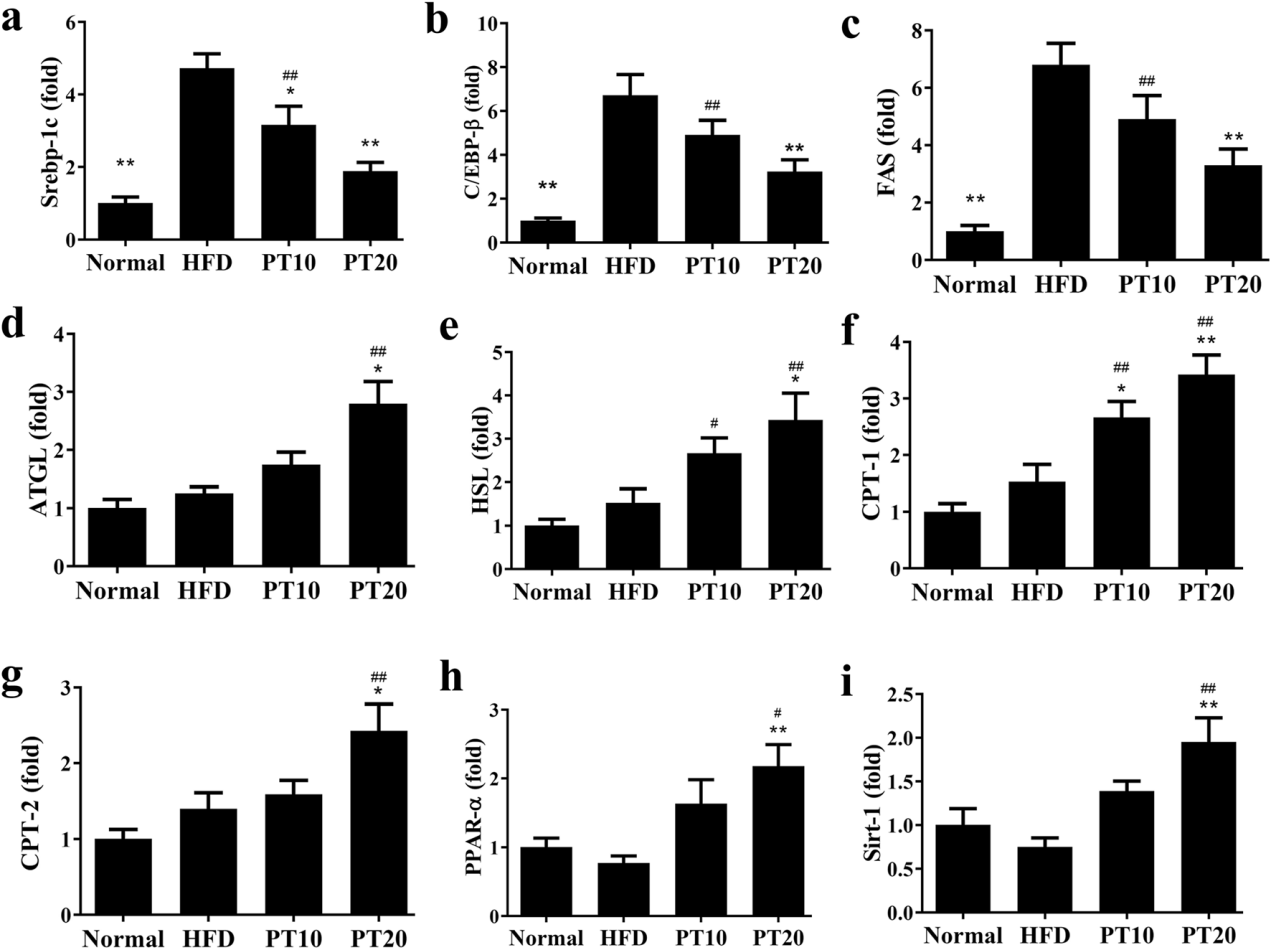
Phloretin (PT) modulated lipogenesis and lipolysis gene expression in liver tissue. **a** Expression levels of Srebp-1c, **b** C/EBPβ, **c** FAS, **d** ATGL, **e** HSL, **f** CPT-1, **g** CPT-2, **h** PPAR-α, and **i** Sirt-1 according to real-time PCR. Fold-changes in expression were measured relative to β-actin expression (internal control). Three independent experiments were analyzed and the data presented as the mean ± SEM; n = 10. *P < 0.05, **P < 0.01 compared to HFD-induced obesity. ^#^*P* < 0.05, ^##^*P* < 0.01 compared to the Normal group

Phloretin suppressed the expression of transcription factors Srebp-1c, C/EBPβ, and FAS compared to the HFD group (Fig. 4a, b). Phloretin enhanced ATGL, pHSL, CPT-1, CPT-2, and PPAR-α expression in mice with HFD-induced obesity (Fig. 4c–f). Phloretin also increased the phosphorylation of AMPK, Sirt1 and phosphorylation of ACC compared to the HFD group (Fig. 4g, h). Furthermore, phloretin significantly reduced TNF-α levels in the serum of mice with HFD-induced obesity (Fig. 4i). Phloretin also decreased TNF-α gene expression in liver and epididymal adipose tissue compared to HFD-induced obese mice (Fig. 4j, k). However, phloretin did not suppress TNF-α gene expression in inguinal adipose of mice with HFD-induced obesity (Fig. 4l). Next, we investigated the expression of genes involved in lipogenesis. Phloretin significantly decreased Srebp-1c, C/EBPβ, and FAS, and increased HSL, ATGL, CPT-1, CPT-2, PPARα, and Sirt1 expression compared to HFD-induced obese mice (Fig. 5).

### Effects of phloretin on serum lipid metabolism

**Fig. 6:**
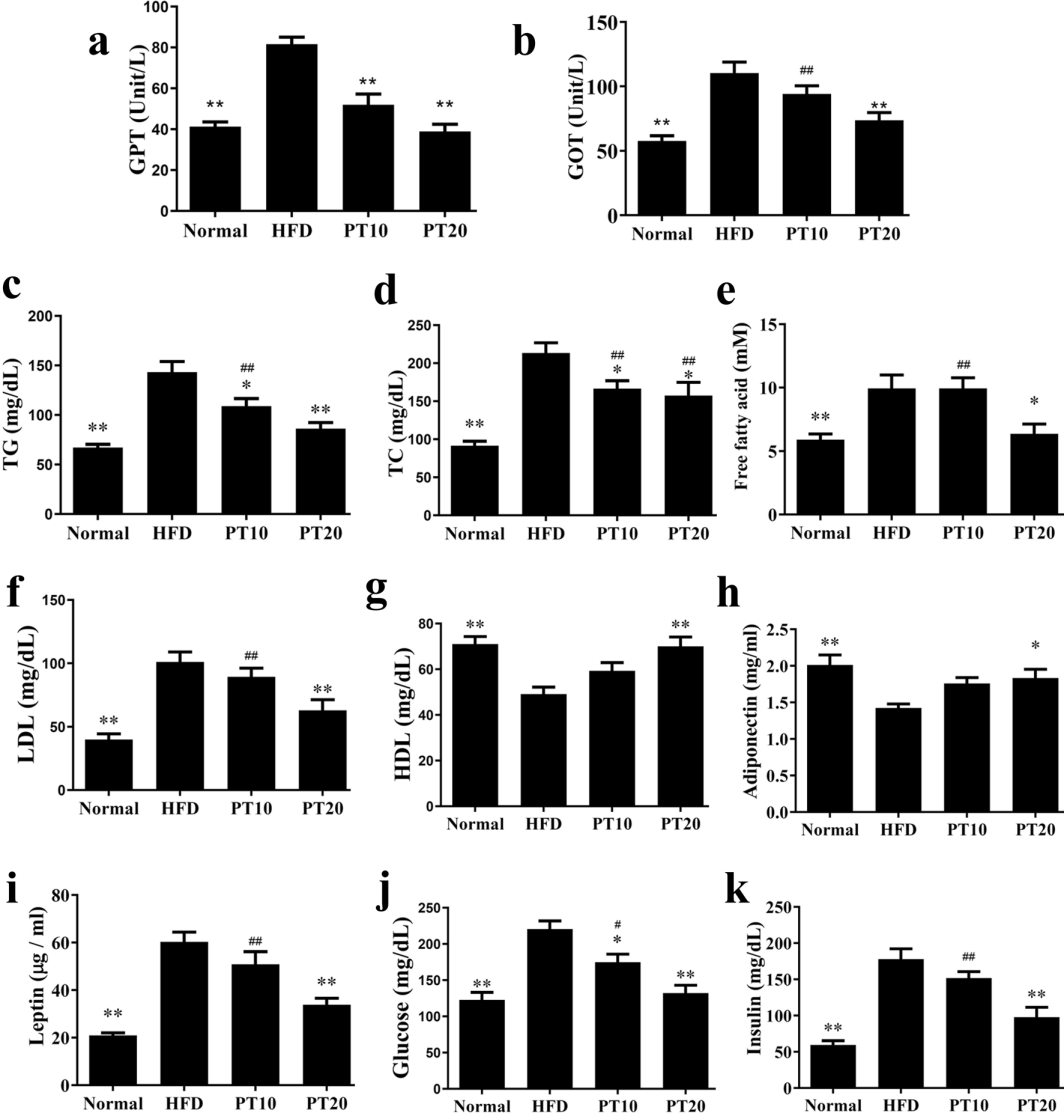
Serum biochemical analysis in mice. **a** Expression levels GPT, **b** GOT, **c** TG, **d** TC, **e** free fatty acid, **f** LDL, **g** HDL, **h** adiponectin, **i** leptin, **j** glucose, and **k** insulin. Three independent experiments were analyzed and the data presented as the mean ± SEM; n = 10. *P < 0.05, **P < 0.01 compared to HFD-induced obesity. ^#^*P* < 0.05, ^##^*P* < 0.01 compared to the Normal group

Phloretin significantly reduced the serum levels of GOT and GPT, recovering liver function in mice with HFD-induced obesity (Fig. 6a, b). Phloretin also significantly suppressed serum free fatty acid, TC, LDL, and TG levels and increased the levels of HDL in HFD-induced obese mice (Fig. 6c–g). We also found that the administration of phloretin significantly inhibited the serum levels of leptin, glucose, and insulin and increased serum adiponectin levels compared to mice with HFD-induced obesity (Fig. 6h–k).

## Discussion

Appropriate exercise and adjusted eating habits may improve obesity and NAFLD [4]. In recent years, scholars have pointed out that some pure plant compounds (e.g., resveratrol, curcumin, and maslinic acid) can reduce weight in obese mice and improve NAFLD, mainly by promoting the Sirt1/AMPK signaling pathway [17, 25, 30]. Our previous study found that phloretin can significantly inhibit the accumulation of oil droplets in differentiated 3T3-L1 adipocytes and significantly inhibit the expression of FAS and adipogenesis-related transcription factors. Phloretin can also improve lipolysis by promoting lipases and phosphorylation of AMPK in 3T3-L1 cells [22]. In recent years, some scholars have found that phloretin can prevent obesity and decrease body weight in HFD-induced mice. However, phloretin did not improve body weight or hepatic lipid accumulation in obese mice [24]. In their treatment model, HFD-induced obese mice were fed for 6 weeks and administered 10 mg/kg phloretin by intraperitoneal injection for only 6 weeks. We speculate that prolonging the phloretin treatment period and increasing the dose of phloretin may have reduced the weight in obese mice. Therefore, we designed an experimental procedure to induce obesity for 16 weeks, and treat mice with 10 mg/kg and 20 mg/kg phloretin twice a week for 12 weeks. The 20 mg/kg of phloretin for 12 weeks effectively reduced the weight of obese mice, as well as the weight and lipid accumulation of epididymal and inguinal adipose tissue.

Obesity is an important risk factor for cardiovascular disease and type 2 diabetes [31]. Long-term excessive intake of a refined diet will accelerate the accumulation of excessive energy in the body and lead to excessive weight [32]. Visceral fat tissue plays an important role in supporting and protecting the organs, but excessive accumulation of visceral fat will surround the organs and affect their function [31]. In mice induced by a HFD, visceral and inguinal adipose tissue accumulate large amounts of TGs, which not only increases body weight, but also increases the adipose tissue weight. Previous studies have found that obese mice do not have reduced adipose tissue weight with intraperitoneal injection of 10 mg/kg phloretin for 6 weeks [24]. However, our experiments found that administration of 10 and 20 mg/kg phloretin by intraperitoneal injection for 12 weeks significantly reduces the weight of epididymal and inguinal adipose tissue in obese mice. Therefore, we conclude that phloretin can not only prevent weight and fat accumulation in obese mice [24], but also improve the adipose tissue weight in obese mice to achieve weight loss.

The liver accumulated more TGs, causing liver steatosis and inducing the development of NAFLD. Fatty liver is defined as excessive accumulation of TGs in liver cells, and the fat content in liver tissue exceeds 5%, or fat vacuole content 10% [33]. The NAFLD score index includes blood biochemical values, fat vacuole number, and macrophage infiltration in liver tissue [28]. The NAFLD score for is significantly higher for HFD-induced obese mice than for normal mice. Phloretin could decrease the liver weight of obese mice, but phloretin did not reduce the ratio of liver weight/ body weight compared to HFD-induced obese mice. We thought that the liver of obese mice increases excessive oil droplets, but liver weight was increased by 1.17 fold in obese mice compared with normal mice. The liver weight was increased by 1.13 fold in obese mice compared with 20 mg/kg phloretin group mice. Hence, the ratio of liver weight to body weight did not significantly decrease in phloretin-treated obese mice. Furthermore, liver cells can transport glucose into liver cells and convert glucose into glycogen to store energy. However, liver cells of obese people accumulate excessive lipids and interfere with energy metabolism [11]. Liver cells will use glycogen to convert into glucose to provide energy for liver cells. Therefore, fatty liver cells will have less glycogen distribution than normal liver cells. In this current study demonstrated that phloretin was able to recover the glycogen accumulation in liver tissue that reduced in HDF-induced obese mice. Hence, phloretin could regulate glycogen synthesis and maintained the metabolic function in the liver. We found that the TG content and number of fat vacuoles in the livers of phloretin-treated obese mice were significantly reduced compared to obese mice. Obese mice treated with phloretin also have significantly reduced serum GOT and GPT values; therefore, phloretin can restore liver function in obese mice. We think that our experimental results confirm that obese mice given phloretin for 12 weeks have significantly reduced NAFLD scores and improved symptoms of NAFLD.

Excessive accumulation of TGs in the liver will cause hepatic steatosis or NAFLD. The activation of transcription factors (including Srebp-1c and C/EBPβ) is important for initiating the expression of genes in the lipid synthesis pathway and activating FAS expression to promote the synthesis of fatty acid chains [34]. Srebp-1c is considered to be the most important transcription factor regulating lipid synthesis [35]. In the current study, we found that lipid accumulation in HepG2 cells and the fatty livers of obese mice can significantly increase Srebp-1c expression. Both phloretin-treated cells and obese mice have significantly reduced Srebp-1c expression and suppressed FAS productions. FAS is an important enzyme for regulating fatty acid chain synthesis and elongation [8]. A previous study found that HepG2 cells transfected with Srebp-1c siRNA and induced with fatty acid did not express Srebp-1c and excessive oil droplets [36]. Therefore, we thought that oleic acid-induced HepG2 cells and the hepatocytes of obese mice would accumulate a large number of oil droplets and excessive TC and TGs, which is closely related to the expression of lipid synthesis transcription factors and FAS. We examined the livers of obese mice, and phloretin significantly reduced the expression of C/EBPβ and Srebp-1c, thereby inhibiting the expression of FAS and the synthesis of fatty acid chains. Therefore, phloretin-treated obese mice have reduced levels of TG and TC in the liver and improved liver steatosis. We used Oil Red O staining to confirm that oleic acid-induced HepG2 hepatocytes have more oil droplets and phloretin can reduce the oil droplet distribution in HepG2 cells. Our cell experiments also found that phloretin has the ability to reduce the expression of Srebp-1c, C/EBPβ, and FAS in HepG2 cells induced by oleic acid. Therefore, we thought that phloretin has the ability to block liver lipid synthesis by inhibiting transcription factors involved in lipogenesis and FAS expression in obese mice.

Liver or adipose tissue from obese mice has decreased AMPK activation [12, 14]. Sirt-1 regulates AMPK expression and induces AMPK phosphorylation [15]. Resveratrol is considered to be a Sirt1 enhancer, and obese mice treated with resveratrol have improved NAFLD and liver steatosis via promotion of the Sirt1/AMPK pathway [37]. AMPK can be used as a sensor of energy regulation to maintain lipid and sugar metabolism in liver and adipose tissue [13]. Previous studies have confirmed that excessive lipid accumulation in the liver and adipose tissue can inhibit AMPK activity and inhibit AMPK substrate ACC phosphorylation, increasing fatty acid synthesis [38]. Thus, reduced AMPK activity would lead to excessive TG accumulation in the liver, accelerating steatosis and NAFLD. In this study, we found that phloretin can effectively regulate the expression of Sirt1 and phosphorylated AMPK in HepG2 cells induced by oleic acid, and stimulate the phosphorylation of ACC to block FAS expression. An assay of liver protein in obese mice provided the same results as phloretin-treated oleic acid-induced HepG2 cells. In addition, HepG2 cells treated with phloretin and AMPK inhibitors also had restored AMPK phosphorylation and inhibited FAS expression. Therefore, our experimental results confirm that phloretin can reduce the accumulation of lipids in the livers of obese mice by regulating the Sirt1/AMPK pathway.

The excessive lipid accumulation of epididymal and inguinal adipose tissue in an obese individual will interfere with organ functions and induce chronic inflammation and dysfunction [39]. Therefore, increasing the breakdown of excessive TG accumulation will significantly improve liver steatosis and weight loss in obese individuals. TGs can be broken down by ATGL into free fatty acids and diglycerides, and activated HSL can break down diglycerides into free fatty acids and monoglycerides [40, 41]. Previous studies have found that phloretin treatment for 6 weeks does not decrease the weight of obese mice [24], but they did not analyze the molecular mechanism of lipogenesis and lipolysis, and we are do not understand the experimental design regarding the expression of lipid metabolism pathways in phloretin-treated obese mice. However, our experiment was designed to administer phloretin for 12 weeks at an increased dose. Interestingly, our experimental results showed that phloretin can significantly regulate the lipid synthesis and lipolysis pathways of the liver. Therefore, phloretin can effectively improve body weight and hepatic lipid accumulation in obese mice. In this study, we also found that phloretin can increase ATGL and phosphorylated HSL expression in oleic acid-induced HepG2 cells. Interestingly, phloretin can also significantly restore ATGL expression when oleic acid-induced HepG2 cells are co-treated with phloretin and AMPK inhibitor (compound C). Therefore, we confirmed that phloretin can increase lipolysis in fatty liver by regulating the phosphorylation of HSL and ATGL and AMPK expression to achieve weight loss and improve lipid accumulation in the fatty livers of obese mice.

Studies have pointed out that the intestinal bacteria of obese people may stimulate inflammation in the liver and adipose tissue through the intestine and circulatory system, and induce more inflamed macrophages to infiltrate the liver and adipose tissue [42, 43]. Bacterial endotoxin and excess free fatty acids could also stimulate macrophages to release more TNF-α to induce insulin resistance of hepatocytes and adipocytes [44]. Therefore, excessive free fatty acids produced by weight loss people need to generate energy through fatty acid β-oxidation, reducing the inflammation in the liver or adipose tissues caused by free fatty acids. In fatty acid β-oxidation, long-chain fatty acids need to be carried by carnitine to enter the mitochondria [7]. CPT-1 and CPT-2 are important enzymes for liver cells, as they bring free fatty acids from the cytoplasm into the mitochondria [45]. Our results demonstrate that phloretin can significantly increase the expression of CPT-1 and CPT-2 in the livers of obese mice, and phloretin increases the production of CPT-1 and CPT-2 in oleic acid-induced HepG2 cells. Interestingly, animal and cell experiments show that phloretin can also enhance PPAR-α expression in the liver tissues of obese mice and HepG2 cells to increase fatty acid metabolism via the β-oxidation pathway. We also found that phloretin can significantly reduce the levels of serum free fatty acids and TNF-α in obese mice. Furthermore, phloretin reduce the levels of TNF-α in the liver and epididymal adipose tissue, improving inflammation and insulin resistance.

Adipocytes in overweight and obese individuals secrete more leptin to affect the hypothalamus and suppress appetite, reducing the accumulation of excessive energy in the body [46]. Our experiments show that obese mice treated with phloretin have reduced serum leptin and increased serum adiponectin. However, the food intake of obese mice treated with phloretin was not significantly different from that of control obese mice. Interestingly, phloretin can regulate fasting blood glucose and insulin levels in obese mice. Previous studies have demonstrated that increasing the levels of adiponectin can effectively reduce insulin resistance in obesity [47]. Therefore, we think that phloretin may improve blood sugar levels by regulating the levels of leptin and adiponectin, improving insulin resistance in obese mice.

Previous researchers have concluded that phloretin can prevent obesity in mice [24]. In the current study, our experimental conclusion demonstrated that phloretin can reduce the body weight of obese mice and the adipose tissue weight. Phloretin could also regulate lipid metabolism by increasing the Sirt1/AMPK pathway, improving liver steatosis in obese mice. Therefore, we think that phloretin has potential as a natural anti-obesity agent for treating NAFLD.

## Data Availability

Please contact the corresponding author for data on reasonable request.

## Abbreviations

ACC-1: Acetyl CoA carboxylase-1
AMPK: AMP-activated protein kinase
ATGL: Adipose triglyceride lipase
C/EBP: CCAAT/enhancer-binding protein
CPT-1: Carnitine palmitoyltransferase 1
CPT-2: Carnitine palmitoyltransferase 2
FAS: Fatty acid synthase
GOT: Glutamate oxaloacetate transaminase
GPT: Glutamate pyruvate transaminase
HDL: High-density lipoprotein
HE: Hematoxylin and eosin
HFD: High-fat diet
HSL: Hormone-sensitive lipase
LDL: Low-density lipoprotein
NALFD: Non-alcoholic fatty liver disease
OA: Oleic acid
PAS: Periodic acid-Schiff
PPAR: Peroxisome proliferator–activated receptor
PT: Phloretin
Sirt1: Sirtuin 1
SREBP-1c: Sterol regulatory element–binding protein 1c
TG: Triglyceride
TC: Total cholesterol
